# Higher unemployment and higher work-related traumatic fatality: trends and associations from the Canadian province of Saskatchewan, 2007–2018

**DOI:** 10.5271/sjweh.4013

**Published:** 2022-04-29

**Authors:** Samuel Kwaku Essien, Catherine Trask, Cindy Feng

**Affiliations:** 1 School of Rehabilitation Science, Faculty of Medicine, University of Saskatchewan, Saskatoon, Saskatchewan, Canada; 2 Division of Ergonomics, School of Engineering Sciences in Chemistry, Biotechnology and Health, KTH Royal Institute of Technology, Stockholm, Sweden; 3 Canadian Centre for Health and Safety in Agriculture, University of Saskatchewan, Saskatoon, Saskatchewan, Canada; 4 Department of Community Health and Epidemiology, Faculty of Medicine, Dalhousie University, Halifax, Nova Scotia, Canada

**Keywords:** Canada, compensation, injury prevention, mortality, occupational health, safety, unemployment, worker

## Abstract

**Objective:**

Although Saskatchewan appears to have the greatest burden of work-related fatality (WRF) in Canada, it is unclear how WRF rates have varied over time. We investigated the WRF rate in Saskatchewan over the past decade and modeled potential risk factors for WRF, including economic indicators.

**Methods:**

In this cross-sectional, population-based study, Saskatchewan workplace traumatic fatalities grouped by year, season, and worker characteristics (eg, age, industry) were used in addition to Statistics Canada labor force survey total employment, total labor force, and the number of unemployed workers by year and season. WRF rates were calculated as fatalities per total number of employed workers. A Poisson generalized additive model was employed to examine the association between WRF rates and personal characteristics, and economic indicators.

**Results:**

The rate remained fairly stable between 2013–2014 and 2015–2017 but sharply increased from 2017 to 2018. On average, the highest rate was observed among workers aged ≥60 years (0.70 ± 0.21 per 100 000). Men had a more than 13-fold greater risk of WRF than women [relative risk (RR)13.7, 95% confidence interval (CI) 10.48–17.9), with the highest RR of WRF observed in the construction industry (RR 9.2, 95% CI 6.1–13.8). The risk of mortality increased non-linearly with increasing unemployment rate, with instability as the unemployment rate reaches the highest modeled values.

**Conclusion:**

Workplace fatality in the province has fluctuated over the past decade, with differential impact observed among industry groups. Furthermore, an increase in the unemployment rate was followed by an increase in mortality risk. Prioritizing and encouraging prevention strategies during periods of economic recessions could help address the incidence of fatalities at work.

Work-related fatalities (WRF) impose a substantial burden on victims’ families, associates, workplaces, and society at large (1, 2). The International Labor Organization reports more than 2.78 million work-related deaths annually (3). Globally, approximately US$3 trillion is spent on work-related injuries and fatalities (4). In Canada, WRF claimed the lives of 1027 workers in 2018, up from 951 fatalities reported in 2017 (5). However, since not all employers and workers are covered by workers’ compensation boards, it is likely the real WFR rate might be higher (6, 7).

In Canada, WRF due to occupational diseases outweighs that of traumatic incidents, with an estimated total of 8906–12 275 versus 972 between 2014–2016 (6). However, occupational disease/illness often has a long latency period, which can complicate prevention efforts; the conditions of exposure and disease development are long past (and often already changed) by the time the WRF is registered. For example, occupational-related respiratory cancer may take several years (often at least ten years) to be detected (8). In contrast, traumatic WRF is often immediately proximate to the workplace incident and the dynamic set of conditions that contributed (9). This proximity could present opportunities for prevention, particularly if aspects of the social, economic, or workplace conditions could be used as leading indicators of increased risk.

Among Canadian provinces, Saskatchewan ranks the highest in WRF with a 5-year rate of 6.3 per 100 000 compared to the Canadian average of 3.4 per 100 000 (10); Saskatchewan rates are higher than the 5.4 per 100 000 observed in the Southeastern United States (considered to be high risk) (11). Although Saskatchewan appears to have the greatest burden of WRF in Canada, it is not clear how these fatality rates have varied over time, or between groups, or with larger societal-level economic shifts. For example, there is evidence that WRF is impacted by a worker’s sex; men have been found to be at higher risk in Taiwan (7.4 versus 0.9 per 100 000) (12), in the United States (6.4 versus 0.6 per 100 000) (13) and in Australia (89% of all youth WRF) (14). Age can also have an impact, with both youngest (12, 14) and oldest (12, 13) categories of workers showing higher risk than the total population. However, it is not clear how different sex and age groups are affected in Saskatchewan.

In addition to an elevated rate of WRF, Saskatchewan has several characteristics that make it uniquely suited to investigating risk factors for WRF. Saskatchewan’s economy is predominantly driven by resource-based industrial sectors such as agriculture, mining, oil and gas, as well as related equipment manufacturing (15). There is evidence that some industrial sectors have elevated risk of workplace fatality, including agriculture (13, 14), transportation (14), mining (12, 13), and primary resources sectors (16).

On a societal level, reliance on resource-based sectors can also make an economy vulnerable to changes in global commodity prices. For instance, Saskatchewan experienced both growth and contraction of economic activity over the years 2008–2018 that impacted provincial GDP and unemployment rate. Employment is an important determinant of health at the individual level, but evidence that macroeconomic measures can predict mortality is mixed. A Finnish cohort study showed that while individual unemployment more than doubles all-cause mortality risk, losing one’s job in a context of unemployment, (ie, from a company with a lot of downsizing) reduces this risk (17). Time-series data from the EU suggests that, when considering individuals both inside and outside the workforce, both higher GDP and higher rates of unemployment have a protective effect leading to decreased all-cause mortality (18). Bonamore et al (19) suggest that the psychological and behavioral responses to unemployment may act differently at different unemployment rates; that is, the factors driving mortality are non-linear. Indeed, they demonstrated that when unemployment rates are as low as 3% each percentage point increase has a protective effect decreasing average mortality by 0.7%. The protective effect decays as unemployment increases, showing no impact at unemployment rates of 17%, and – at rates of 25% – each percentage point increases average mortality by 0.4% (19). Very little of the research on macroeconomic effects have investigated the impact on work-related fatalities, but it seems clear that such a complex relationship could result in non-linear effects. At present, the paucity of studies incorporating economic indicators in WRF modeling has limited understanding of how indicators of economic up- and downturn impact WRF; this correspondingly limits the ability to use economic factors as leading indicators to target prevention activities.

Saskatchewan’s environmental conditions could also impact WRF risk. Seasonal extreme temperature (defined as the top and bottom 25% of annual observed temperatures) have been shown to increase risk of work-related traffic crashes in Italy (20). Situated on the Northern prairie, Saskatchewan’s arid climate has an annual temperature swing of over 70 degrees Celsius, with lows reaching -40 °C in the winter and summer highs of +35 °C. This provides a contrast in environmental conditions by season that allows for investigation of the unique hazards related to winter weather, which can include cold exposure (and related potential complications with personal protective equipment), and transportation risks (21), including transport to emergency care.

To address the current gaps in the literature and make use of the unique research context provided by this setting, this study aims to investigate work-related fatality rate in Saskatchewan over the past decade by: (i) determining the nature of time trends (monotonic, constant, increasing or decreasing) overall and by industrial sector, and (ii) modeling potential risk factors for WRF, including economic indicators.

## Methods

### Data sources and key variable description

Data from Saskatchewan Workers Compensation Board (SK-WCB) and Statistics Canada labor force survey (LFS) from 2007–2018 were used (22). The number of traumatic WRF were extracted/retrieved from the SK-WCB data file using the diagnosis summary description and were grouped by year, seasons or quarters, age, sex (male/female), and industry sector. Traumatic WRF are defined here as those designated ‘0’ on the claims variable ‘occupational disease’; all such accepted fatality claims that occurred among persons aged ≥15 years were included in this study. In addition, total employment, total labor force, and the number of unemployed workers all by year and quarters were retrieved from the LFS (22).

The age variable was categorized into six age groups (15–19, 20–29, 30–39, 40–49, 50–59, and ≥60 years). Also, 12 industries included in the current study were further categorized into three broad groups for a visualization based on the level of proneness to WRF (industries with higher fatality rates: construction, transportation, and warehousing, mining; industries with middle fatality rates: business, public administration, professional, manufacturing; and industries with lower fatality rates: healthcare, wholesale, accommodation, agriculture, and education). However, these groups were recategorized into seven groups (construction, transportation, and warehousing, mining, business, professional, manufacturing, and other) for modeling purposes. More specifically, those industries that are consistently below 1 fatality per 100 000 over the study period and as well cases with reported unknown occupation code were combined into a single category ‘other industries’. Cases with unknown occupation codes were also added to the ‘other’ category to help address potential modeling issues related to missing data. Temporal effects were assessed using seasons/quarters (quarter 1: January-March; quarter 2: April-June; quarter 3: July-September and quarter 4: October-December).

For assessing the impact of economic indicators on WRF, the following economic variables were extracted from the LFS: provincial unemployment rate and provincial gross domestic product (GDP). The unemployment rate was derived from the total quarterly unemployed workers per total quarterly labor force in Saskatchewan during the study period. The unemployment rate was further categorized into three rate groups (group 1: 3.2–4.4%; group 2: 4.5–5.2%; and group 3: 5.3–6.8%) based on the distribution of the calculated unemployment rate. Due to the unavailability of quarterly GDP estimates, yearly Saskatchewan GDP (23) was replicated for all quarters in each study year.

### Data analysis

WRF rates were calculated using the number of fatalities in a given age group, season, and industrial sector as the numerator and the total number of employed workers in the corresponding group as the denominator. The calculated rate was expressed per 100 000 population. A generalized additive model (GAM) (24) with a Poisson distribution was employed to examine the association between WRF rates and personal characteristics, including age, sex, seasons, industrial types, and economic indicators. GAM is a flexible statistical technique that captures the nonlinearities in continuous covariates via the use of a smoothing function (24, 25). For the continuous variables such as age and year of fatality, B-spline functions were used to flexibly model their potentially nonlinear effects on the risk of fatality. Moreover, the effect of the economic indicators such as the unemployment rate may not act simultaneously on the fatalities, and the effect is most likely somewhat delayed. As a result, four lags were evaluated (lags 0, 1, 2, and 3 seasons), and the lag with the lowest Akaike Information Criterion (AIC) was selected.

In the process of building the multivariable models, variables with P-value <0.25 in the univariate regression were retained for further consideration. Manual backward model selection was applied to select significant variables with P-value <0.05 for the final model. To test if the time trend differs by age groups, gender groups, or age-by-gender groups, and industrial sectors, interactions between variables were examined. Also, overdispersion was evaluated by comparing Poisson, and negative binomial (NB) regressions were performed; the results indicated that Poisson and NB performed equivalently according to the AIC, so no overdispersion relative to Poisson was identified. Additionally, the analysis of deviance was employed to further examined the model’s fit. The study size was arrived at via collapsing/ aggregating traumatic fatality cases by 12 years by 4 seasons by 11 age-group categories by 7 industrial sectors by sex, which yielded a sample size of 7392 fatality rate estimates for modeling. All GAM-related analysis was performed in R-software (*mgcv* package).

## Results

### Descriptive

The study identified 220 traumatic WRF cases from 2007 to 2018. Of these, 206 (93.6%) were among male claimants, and 14 (6.4%) among female. Persons aged ≥60 years constituted 26.3% of the total WRF cases, followed by those aged 50–59 years (21.3%) and 20–29 years (20.5%). Three industries contributed to the majority (51.4%) of WRF cases: (construction 55 (25.0%); transportation and warehousing 34 (15.5%); and mining 24 (10.9%) during the study period.

### Trend analysis

**Figure 1:**
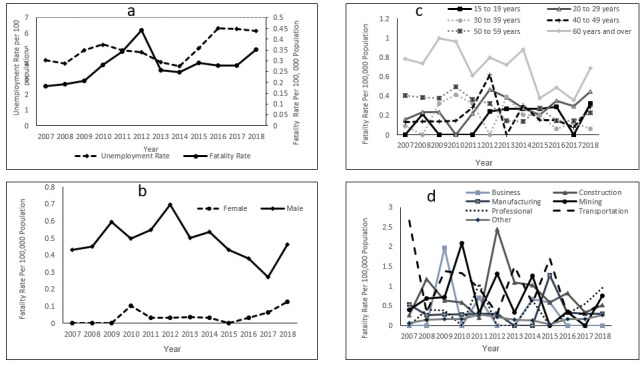
Work-related traumatic fatality crude rate in the Canadian province of Saskatchewan, 2007–2018 by age, sex, industrial sector and unemployment rate.

Figure 1(a) illustrates the overall WRF rate trends in the province of Saskatchewan from 2007 to 2018. The average WRF rate during this 12-year period was 0.28 ± 0.07 per 100 000. The highest annual WRF rate occurred in 2012 (0.44 per 100 000), while the lowest occurred in 2007 (0.19 per 100 000). The overall WRF rate remained fairly stable between 2013–2014 and 2015–2017, then sharply increased from 2017 to 2018. Unemployment shows similar variation over this period, suggesting evidence of a time lag before the impact on fatalities; unemployment goes up first, especially between 2007 and 2010, whereas fatalities go up later between 2015 and 2018.

Figure 1(b) shows differences in WRF rates between female and male workers between 2007 and 2018, with consistently higher WRF rates among males. For female workers, a zero fatality rate was observed between 2007 and 2009, a stable rate between 2011 and 2014, and finally an increasing trend between 2015–2018. Although the rate among males showed a prolonged swift decline between 2014 and 2017, this pattern was reversed by a sharp rise between 2017 and 2018, consistent with the trend among females during the same period. While the highest female rate occurred in 2018 (0.13 per 100 000), the highest male rate rather occurred earlier in 2012 (0.69 per 100 000).

Figure 1 (c) outlines the trends in WRF rates stratified by workers’ age during the years 2007–2018. The results show that WRF rates in those aged ≥60 years were consistently higher for each study year when compared to the other age groups. Also, stable WRF rates were observed among those aged 50–59 years between the study years 2007–2009, 2013–2014, and 2016–2017 but rose the year after. Moreover, WRF rates also remained stable between 2007–2010 for those aged 40–49 years and between 2012–2016 for those aged 15–19 years. The oldest age categories, 50–59 and ≥60 years, recorded nonzero fatality rates in all years under study; all younger categories had at least one year with zero fatalities during the study period. On average, the highest rate was observed in those ≥60 years (0.70 ± 0.21 per 100 000), followed by those aged 50–59 years (0.29 ± 0.12 per 100 000). The third highest WRF rate was reported among those aged 20–29 years (0.27 ± 0.13 per 100 000) while the lowest was reported among those aged 15–19 years (0.16 ± 0.14 per 100 000). The average WRF rates among those aged 30–39 years and 40–49 years were 0.18 ± 0.14 and 0.20 ± 0.16 per 100 000, respectively.

Figure 1(d) presents the trends in WRF rates from 2007 to 2018 stratified by industry. No industry consistently experienced a higher WRF rate for all years during the study period. In addition, there were unique years for which each industry experienced its highest WRF rate. The transportation and warehousing industry experienced its highest rate in 2007, construction in 2012, mining in 2010, business in 2009, manufacturing in 2015, professional in 2018, and the ‘other industries’ category in 2011. Except for two industry groups (construction & transportation and warehousing) that experienced non zero fatality during all study years, the remaining industries had ≥1 zero fatality rate. Furthermore, trend results show that since 2017, five out of the seven industrial groups are on increasing trends. These include construction, mining, professional sector, transportation and warehousing, and the other industries category. Manufacturing and business rates have remained stable since 2016. On average, the highest rate was observed among those in the transportation and warehousing industry (0.99 ± 0.74 per 100 000), followed by the construction industry (0.80 ± 0.60 per 100 000) and the mining industry (0.68 ± 0.61 per 100 000). The fourth industry was the professional sector (0.36 ± 0.38 per 100 000), followed by the manufacturing sector (0.35 ± 0.33 per 100 000) then the business sector (0.33 ± 0.59 per 100 000). The ‘other’ category reported the least WRF rate (0.16 ± 0.07 per 100 000) in the province.

### Generalized additive model

Table 1 presents the AIC scores comparing negative binomial and Poisson models and deviance residual comparing various Poisson models. The model evaluation based on the AIC revealed that the Poisson generalized additive model using unemployment rate lagged by one quarter yielded the best fit. A further comparison of the various Poisson models via the residual deviance analysis showed that the Poisson generalized additive model with unemployment rate lagged by one quarter compared to all the various Poisson models yielded the smallest residual deviance. Hence, based on the findings from table 1, all subsequent analyses were carried out using the Poisson generalized additive model with unemployment rate lagged by one quarter.

**Table 1:** AIC scores for the negative binomial and Poisson generalized additive models for modeling the effects of personal characteristics, economic factors on work-related fatality risk, Chi-square test based on the deviance residual is used for comparing the model fits among various Poisson models. The minimum values are **bolded** to indicate model performance

Lag	AIC		Analysis of Deviance		
	
	Negative binomial	Poisson	Deviance residual*	Difference in deviance residual	Pr(>Chi)
0	6697.29	6689.29	4905.4	lag 0 vs. lag 1	6.213e-07
1	6674.56	6669.24	4884.3		
2	6714.21	6706.61	4922.4	lag 2 vs. lag 1	7.365e-12
3	6741.33	6711.48	4931.1	lag 3 vs. lag 1	3.508e-11
4	6736.21	6732.74	4967.0	lag 4 vs. lag 1	4.892e-14

Table 2 reports the effects of personal characteristics and economic factors on WRF risk. The results revealed that gender is significantly associated with WRF, with a relative risk (RR) of 13.68 [95% confidence interval (CI) 10.48–17.86] times higher risk among males than females. In terms of the seasonal effect, compared to Quarter 4 (October-December or the autumn), the RR of fatality is significantly lower in Quarter 2 (April-June or the spring) (RR 0.69, 95% CI 0.57–0.83). No significant difference was identified between Quarter 1 (January-March or winter) and Quarter 4 (RR 0.84, 95% CI 0.64–1.11), and Quarter 3 (July-September or summer) and Quarter 4 (RR 0.90, 95% CI 0.74–1.10). The industrial sectors were found to be significantly associated with WRF with P<0.001. More specifically, in comparison to the business industry, the industry with the highest RR of WRF was the construction industry (RR 9.2, 95% CI 6.1–13.8), followed by transportation and warehousing, mining, and then professional. The lowest RR of WRF was the manufacturing industry (RR 2.06, 95% CI 1.28–3.30).

**Table 2:** The estimated effects of study participants’ demographics and province-level economic factors on work-related fatality risk. [Cl=confidence interval; EDF=effective degrees of freedom; SE=standard error; RR=relative risk]

Covariate	Estimated covariate effects			

	Estimate	SE	RR	95% CI
Gender				
Female (ref)				
Male	2.616	0.136	13.68	10.48–17.86
Quarters				
Quarter 4 (Oct-Dec) (ref)				
Quarter 1 (Jan-March)	-0.170	0.139	0.84	0.64–1.11
Quarter 2 (April-June)	-0.372	0.097	0.69	0.57–0.83
Quarter 3 (July-Sept)	-0.103	0.099	0.90	0.74–1.10
Industry group				
Business (ref)				
Construction	2.218	0.208	9.19	6.11–13.82
Manufacturing	0.722	0.240	2.06	1.28–3.30
Mining	1.380	0.221	3.97	2.58–6.13
Professional	1.063	0.229	2.90	1.85–4.54
Transportation and warehousing	1.509	0.218	4.52	2.95–6.94
Other industries	2.411	0.206	11.14	7.44–16.70
Approximate significance of smooth terms				
Smooth covariate	EDF			
Age	8.141			
Year	7.578			
Unemployment rate ^a^	8.856			

a Unemployment rate lagged by one quarter.

**Figure 2:**
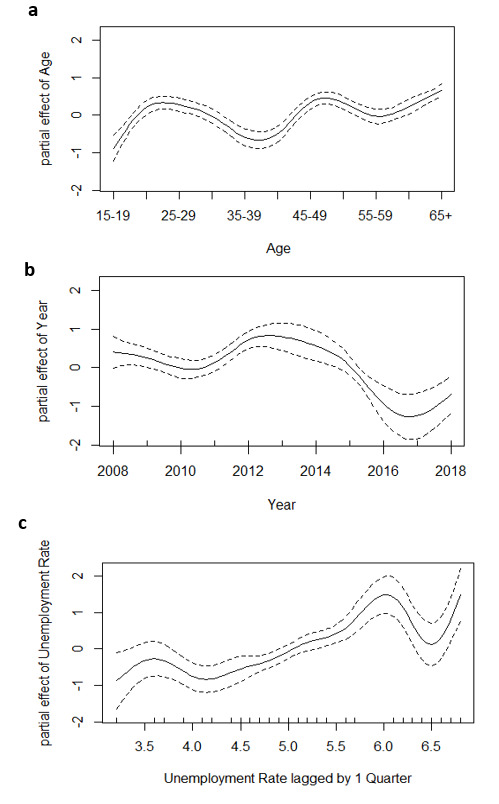
Nonlinear effects of age, year, and unemployment rate (lagged by one quarter) on the logged work-related fatality (WRF) risk. The solid lines represent the estimated non-linear effects, and the dashed lines represent the corresponding 95% confidence intervals of the non-linear effects.

Figure 2 presents the effects of the continuous covariates on the WRF. Fig 2(a) shows the nonlinear effect of age on WRF risk with age groups 25–29, 50–54, and ≥60 years having a higher risk of mortality than the rest of the age groups. Fig 2(b) reveals that the risk of WRF slightly declined from 2008 to 2011, followed by an increasing trend and peaked in the year of 2012, and then declined afterward to 2017 then slightly increased again in the year of 2018. Fig 2(c) indicates that the risk of mortality significantly increased as the unemployment rate increases, with instability as the unemployment rate reaches the highest modeled values.

We evaluated the model fit by treating the unemployment rate as a categorical variable with three levels determined by its quartiles. The resulting model did not fit as well as the model that flexibly modeled the nonlinear effect of the unemployment rate as a continuous variable using spline function (AIC=7022.1 for categorical unemployment versus AIC=6968.5 for flexibly modeled continuous unemployment). There was also no appreciable difference in the shape of the line or CI between the two models. Since the two models are consistent in terms of the impact of unemployment, we conclude that the shape of the spline is not an artifact due to the disadvantages of the spline method. As a result, we present only the final model using unemployment as a flexibly modelled continuous variable.

## Discussion

Understanding time-trends of disease (including mortality and morbidity rates) paves the way for deciding if there are justifiable grounds to carry out a deeper investigation into an underlying condition (26). In the 12-year period covered by our analysis, the rate of WRF in the province fluctuated; there was a fairly stable rate observed between 2013–2014 and 2015–2017, but sharply increased from 2017 to 2018.

We found that the risk of mortality significantly increased as the unemployment rate increased, although this relationship was nonlinear with a drop when the unemployment rate is at the highest observed levels of 6.5%. While there are very few similar studies which can serve as direct comparison, this finding seems unique. Recessions and higher unemployment have generally been found to decrease the rate of overall work-related injuries (ie, not WRF) (27). Higher unemployment rates at a societal level have also been found to reduce the tendency of individual unemployment status to increase all-cause mortality (28) There are several proposed mechanisms for a protective effect of higher unemployment rates. Boone and Ours (29) call the most common pattern ‘pro-cyclic’, where economic growth (and low unemployment) linking low unemployment to ‘boom time’ mechanisms that increase exposure to hazards. For example, boom time incentives for high production may lead to more overtime work and concomitant fatigue, and may result in older, less safe equipment being pressed into service by established enterprises or by smaller new firms (27). Low unemployment and elevated demand for labor may induce the hiring of more young and/or inexperienced workers into an industry; these workers may lack the training, experience, and self-efficacy to recognize, report, and mitigate hazards (27). Economic recessions typically lead to higher unemployment levels in the more hazardous industries, such as construction, and consequently may result in lower overall work-related injury rates (27). However, the current study also tested the interaction between industrial sectors and time but was found not be statistically significant. Thus, it does not appear that unemployment rate has a differential effect in specific industries – again defying much of the other proposed ‘pro-cyclic’ mechanisms for workplace injury.

In contrast, our findings demonstrate what Boone and Ours call a counter-cyclic pattern (29), with recession/higher unemployment showing increased workplace risk. Given the apparent contradiction, it is possible that many or all the previously-proposed pro-cyclic mechanisms linking lower unemployment to higher risk of workplace *injury* may not apply in the same way to fatality. The few available studies that link macroeconomic factors with fatality typically do so for all-cause mortality in the general population (17–19); ‘diluting’ WRF with all other fatalities would introduce a host of other mechanisms and driving forces, including psychological responses to job loss (eg, substance abuse) (30), which limits the applicability to traumatic workplace fatalities. It should also be noted that much of the work cited here, and particularly that informing proposed mechanisms, does not include the bust and recovery cycle of the Great Recession that started in 2008. In fact, much of the mechanistic theory draws on research on the Great Depression of the 1930s (27); clearly the 80 intervening years leading up to the current studied period saw tremendous changes in the context for both labor and occupational health and safety regulations, enforcement, and practice.

The explanation for this seeming contradiction between the counter-cyclic pattern seen in our results and more commonly reported pro-cyclic pattern may lie in a combination of worker empowerment and employer capacity to respectively flag and mitigate workplace hazards. It has been suggested that in periods of high unemployment, workers defer making claims (27, 29); this suggests that the true injury rates are underrepresented during high unemployment due to fear of job loss. This effect is reduced with more serious injury (27) and since workplace fatalities are difficult to ignore or under-report they might be expected to be unaffected, removing this mechanism entirely for this outcome. The fear of job loss in a hostile labor market may also extend to reducing workers’ willingness to voice concerns about hazards that could increase their risk of fatality. In Canada all workers have the right to refuse unsafe work without fear of reprisal, but there is an acknowledged risk in doing so (48). A study of young Canadian workers indicated that despite knowing of this right, workers (from a sample including non-unionized, part-time, and precarious employment) expressed reluctance to exercise it (48). Indeed, perceived job insecurity has been shown to decrease worker safety motivation and compliance among food processing workers (49), and economic recession had a temporary effect on maritime crew’s motivation and intention to comply with the safety regulations (50). Such decreased motivation would be expected to include decreased hazard reporting. Even if hazards are reported, economic constraints may incentivize an employer to deprioritize occupational health and safety actions. A review found that 73% of included studies from 1966 to 2007 reported that downsizing/restructuring and job insecurity were related to poorer occupational health and safety outcomes (51). Although only five of the studies assessed injury and none assessed fatality, the consistency of the findings across a diversity of jurisdictions, study designs, and outcomes suggests a pattern that is both compelling and intuitive (51). For example, as a result of rationalization the occupational health and safety tasks may be re-delegated to a generalist who has little time or OHS-specific training. There is less time for risk assessment, and even when there is worker reporting or advocacy around hazards, there is limited time allocated to mitigating them.

Our study found significantly higher WRF rates in some industrial sectors; in comparison to the reference category ‘business sector’, the industry with the highest relative risk (RR) of WRF was the construction industry (RR 9.2, 95% CI 6.1–13.8), followed by transportation and warehousing (RR 4.5, 95% CI 2.9–6.9), then mining (RR 3.9, 95% CI 2.6–6.1). There were no significant interactions between industrial sector and time. These are consistent with other regions of Canada; in the Canadian province of Ontario, the construction industry constituted only about 8% of the province’s labor force (31, 32), but nonetheless reported the highest number of traumatic WRF in 2017 (31). From 2011 to 2017, the construction industry consistently reported the highest traumatic WRF in Ontario, followed by the transportation industry and the mining sector (33). The risk of traumatic WRF is expected to be highly dependent on the nature of the tasks and corresponding hazards to which workers are exposed; high risk tasks and exposures are anticipated to occur at greater frequency within high-risk industries.

Consistent with previous literature, we found worker factors of age and sex to be significantly related to WRF risk. On average, the highest rate was observed among those ≥60 years (0.70 ± 0.21 per 100 000) and in the transportation and warehousing industry (0.99 ± 0.74 per 100 000), followed by the construction industry (0.80 ± 0.60 per 100 000). Our finding that older persons were at increased risk of WRF compared to other age categories is consistent with the literature on this topic (34–36). It has been suggested that increasing risk in older age is related to age-related declines in physical capacities important to prevent traumatic incidents (36), for example reduced hearing, vision, balance, and reaction time. Studies in high-risk industries like agriculture provide an example of these declines are especially problematic when operating heavy machinery. For example, detailed investigation of hospital records and coroner’s reports show that farmers aged ≥60 years had an age-adjusted mortality rate of 32.8 per 100 000 workers, three times that of younger farmers, and they most often died while performing farm tasks involving machinery (52). A review by the same authors demonstrated increased risk with medication use, disability, and co-morbidities such as previous injury, hearing problems, depression, arthritis, and sleep deprivation; these risk factors also contribute to falls and motor vehicle collisions in nonfarm populations (53).

In the GAM model, men had more than 13-fold greater risk of WRF than women (RR 13.7, 95% CI 10.48–17.9). This is also consistent with the literature, where male workers have been consistently shown to be at higher risk of a workplace fatality (34, 35, 37). It should be noted that in this report we use the sex-based coding ‘male–female’ as per the WCB data source. However, in this secondary analysis it is not possible to disentangle the effects of sex and gender, and it is likely that much of the apparent “sex differences” are in fact a gender-related difference driven by differential gender representation by industry, occupation, and even task delegation within departments and job titles. There is evidence that women consistently do more of the monotonous and repetitive work (38, 39), and men take on tasks that include more exposure to life-threating safety hazards (40). Even with the very progressive gender employment policies in Finland, there remain substantial gender segregation within industries and occupations (41). It is of note that since 2015 our data show a steady increase in the fatality rate for women; this may represent the start of an increase in risk for women, since women continue to increase participation in traditionally male-dominated professions (42). The only seasonal difference showed spring (Quarter 2, April-June) (RR 0.69, 95% CI 0.57–0.83) to have significantly lower WRF than autumn (Quarter 4, October-December). In other regions both warm and cold weather seasons have been shown to contribute to WRF, especially in the construction industry (43, 44). Our findings do not seem to link directly to winter-weather risk since the coldest months of January and February are not included. However, the these ‘shoulder seasons’ may experience more thaw-refreeze cycles that result in more ice, potentially increasing risk of falls and vehicle collisions.

### Methodological considerations

Fatality rate estimation and its interpretation are highly dependent on the population coverage (denominator) used [eg, full-time employment (FTE) hours and labor force survey (LFS)] (45, 46). Although the advantages of using FTE as a denominator for WFR is reported in the literature (45), FTE does not capture workers of self-insured employers in some Canadian provinces, including Saskatchewan (45, 47). In contrast, LFS turns to include both employees covered by a compensation board and those with self-insured employers (45, 47), but may be subject to errors due to sampling (34). As an additional consideration, the time lag between unemployment and fatality in the present study was one quarter (three months) and was selected based on model fit criterion. However, it is worth revisiting different latencies in future studies, since it may take a longer time for the effect of the unemployment rate to manifest.

### Strengths and limitations

This is the first study in the province to have explicitly explored the impact of economic indicators in association with workplace fatality rate and adjusted for other individual and employer’s level factors using smoothing spline techniques. Moreover, the study also used 12 years of data, which is also quite rare in the occupational health literature in Canada. Although the use of the LFS as a denominator captured a representative sample of both self-insured employees and those covered by the Saskatchewan workers compensation board, errors due to sampling could arise.

### Concluding remarks

Over the past 12 years, the rates of workplace fatality in Saskatchewan have fluctuated, with a rising trend observed between 2017 and 2018. Over this time period, unemployment also fluctuated and a significant, non-linear relationship was identified with WRF; risk of mortality significantly increased as the unemployment rate increased from 4.0% to 6.0%, with instability as the unemployment rate reaches the highest modeled values (6.0%–7.0%). Construction, mining, transportation and warehousing accounted for the majority of the fatalities. The disparities of workplace fatalities among industry groups highlight the need for more industry-specific prevention efforts. Hence, prioritizing and encouraging prevention strategies during periods of economic recessions could help address the incidence of fatalities at work.
